# Highly Efficient Chlorine Fixation Based on Organic Selenium for 3.7‑V Aqueous Batteries

**DOI:** 10.1021/jacs.5c16178

**Published:** 2026-03-16

**Authors:** Ze Chen, Yiqiao Wang, Zhiquan Wei, Ao Chen, Xinliang Li, Zhaodong Huang, Shixun Wang, Chunyi Zhi

**Affiliations:** 1 Wu Jieh Yee School of Interdisciplinary Studies, 34743 Lingnan University, 8 Castle Peak Road, Tuen Mun, Hong Kong 999077, China; 2 Department of Materials Science and Engineering, 53025 City University of Hong Kong, 83 Tat Chee Avenue, Kowloon, Hong Kong 999077, China; 3 School of Physics and Laboratory of Zhongyuan Light, 12636 Zhengzhou University, Zhengzhou 450052, China; 4 Department of Chemical and Biological Engineering, 58207 Hong Kong University of Science and Technology, Clear Water Bay, Kowloon, Hong Kong 999077, China; 5 Department of Mechanical Engineering, 25809 The University of Hong Kong, Pokfulam, Hong Kong 999077, China

## Abstract

The limited high-potential cathode materials for aqueous batteries have hindered their improvement in energy density improvement. Chlorine-based batteries with Cl^0^ to Cl^–^ redox reaction (ClRR) are promising for high-performance aqueous batteries due to their high redox potential and large theoretical capacity. However, the inherent gas–liquid conversion feature of ClRR and poor Cl fixation can cause Cl_2_ leakage, reducing battery reversibility and raising safety concerns. Herein, we utilize a Se-based organic molecule, polymerized benzoselenadiazole (poly-PhSe), as the Cl-anchoring agent and realize an atomic level-Cl fixation through chalcogen-halogen coordinating chemistry, achieving a highly reversible ClRR with extra-low Cl_2_ emission and a notably high-discharge voltage (3.7 V when paired with a graphite anode). The promoted Cl fixation and multivalence conversion of Se contribute to a three-electron conversion process, resulting in a significantly high-discharge capacity of up to 344 mAh g^–1^ with an average output voltage of 1.79 V and a high Coulombic efficiency of 99.1%. Based on the superior reversibility of the developed poly-PhSe electrode with ClRR, a remarkable rate performance and cycling performance (with a capacity retention of 84.6% after 850 cycles) are achieved. Significantly, the pouch cell delivers a record areal capacity of up to 5.3 mAh cm^–2^, demonstrating great potential for practical applications. This chalcogen-halogen coordination chemistry between the Se electrode and Cl provides new insight for developing reversible and efficient batteries with halogen redox reactions.

## Graphical abstract

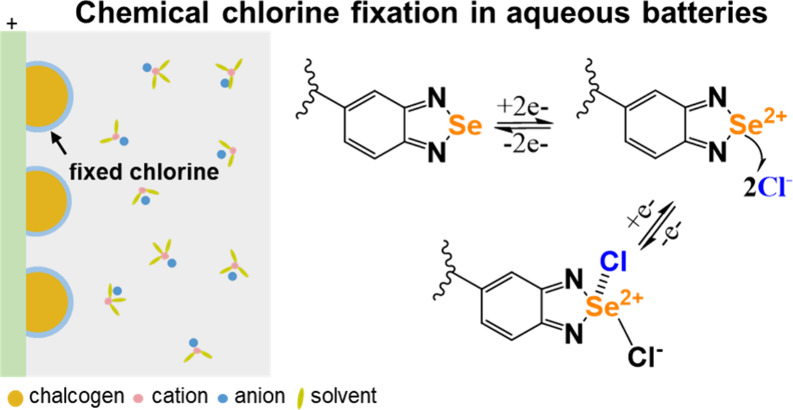

## Introduction

Aqueous batteries are promising for solving the safety issues of modern lithium-ion batteries, and other advantages such as low cost and environmental friendliness attract more research efforts.^1,2^ However, the limited cathode materials (most are transition-metal-oxide cathodes) and the unstable aqueous electrolytes have precluded higher energy densities.^3^ Many strategies have been developed to expand the electrochemical window of aqueous electrolytes, including “water-in-salt” electrolytes,^4^ organic additives,^5^ and decoupling aqueous electrolytes.^6^ Conversely, there has been limited progress in developing high-performance aqueous cathodes. Chlorine redox reaction (ClRR) is capable of working in aqueous environments, delivering a high reaction potential (1.36 V versus standard hydrogen electrode (SHE)) and outstanding theoretical gravimetric capacity (756 mAh g^–1^, for one-electron Cl-based conversion reaction).^4,7,8^ However, the development of ClRR electrodes has been hindered principally due to the poor reversibility of Cl_2_-based batteries and severe Cl_2_ leakage.^9,10^ Therefore, improving Cl fixation is essential for fabricating reversible and environmentally friendly Cl_2_-based batteries. Recently, ClRR reversibility has been enhanced through confining oxidized Cl inside adsorption-type host materials, such as activated carbon (AC), graphite, and porous carbon spheres.^11,12^ Although the physical adsorption of Cl_2_ on carbon-based materials could retard the release of toxic Cl_2_ gas to some extent, Cl_2_ evolution could not be fully eliminated, impairing Coulombic efficiency (CE) of ClRR (Figure 1a).^11,13,14^

**Figure 1:**
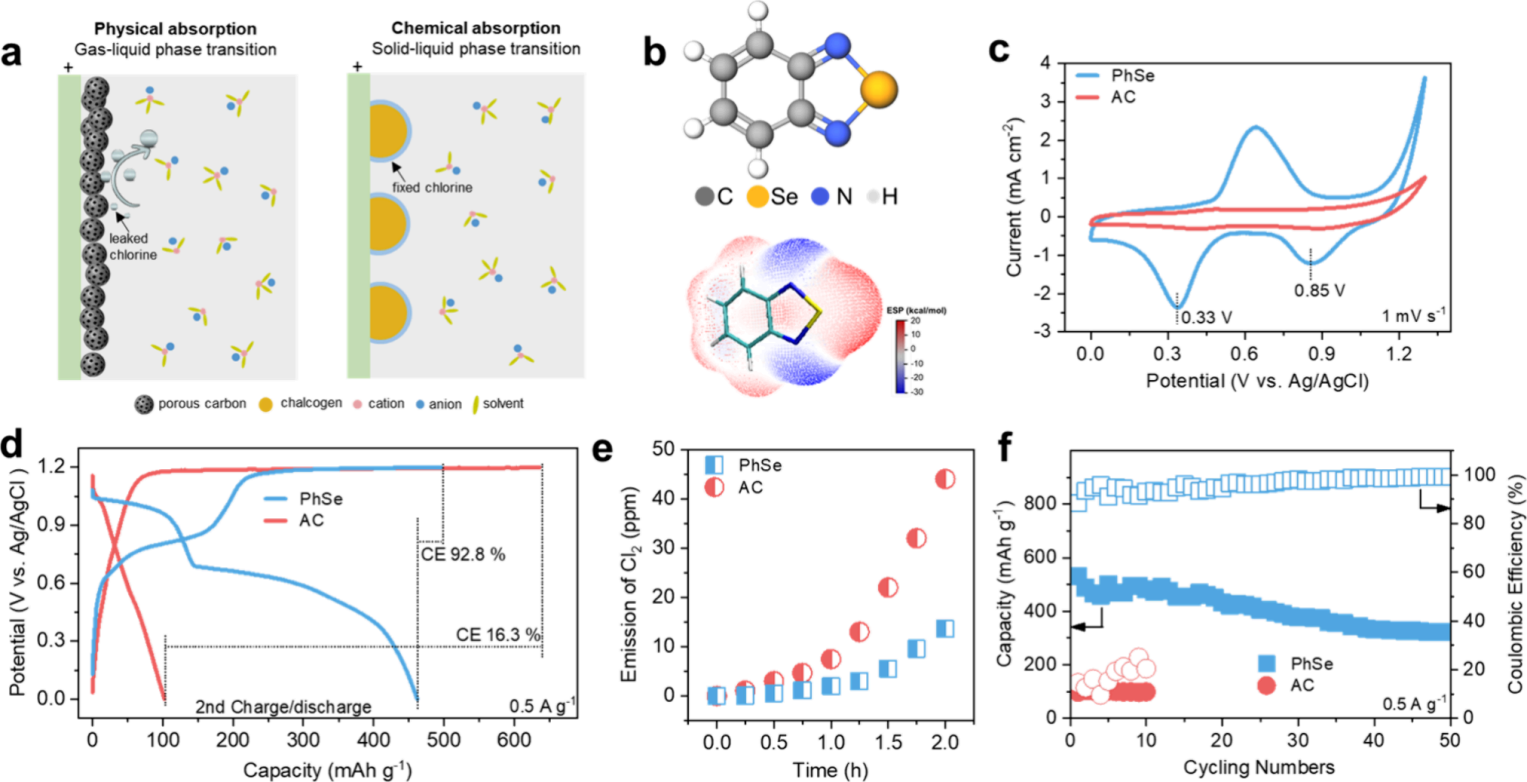
Optimized chlorine fixation based on a chemical absorption strategy with organic Se. (a) Schematic illustration of the physical adsorption and chemical adsorption strategies for chlorine fixation; (b) molecular structure and the corresponding surface potential energy of PhSe; (c) CV curves and (d) GCD profiles of the AC electrode and PhSe electrode; (e) gaseous Cl_2_ evolution during the charging process of the AC electrode and PhSe electrode; (f) long-term stability of the AC electrode and PhSe electrode at 0.5 A g^–1^.

Despite inferior Cl fixation by physical absorption, exploring potential strategies to anchor Cl^0^ at the electrode side is encouraging for pursuing highly efficient ClRR.^4,15,16^ In principle, oxidized Cl^0^ would be stored in a solid state at the electrode side by strong chemical bonding with the anchoring agent (Figure 1a).^13,17,18^ Since Cl^0^ with strong electrophilicity would spontaneously produce gaseous Cl_2_ molecule, anchoring agents should be more energetically favorable to bind with Cl^0^ and prevent generation of Cl_2_ molecules.^19^ Bromine, iodine, and selenium were exploited as Cl-anchoring agents. The formation of interhalogens BrCl and ICl and the chalcogen-halogen compound Se–Cl during cycling delivered a reversible charge storage process, implying reliable Cl fixation by chemical-bonding strategies.^13,20,21^ In addition, the intrinsic valence diversity of Se can facilitate redox kinetics during the charging/discharging process.

Herein, we explore a Se-based organic molecule, polymerized benzoselenadiazole (poly-PhSe), as the anchoring agent for Cl fixation based on chalcogen-halogen coordination chemistry. Highly reversible ClRR is obtained in the poly-PhSe electrode, and when paired with a graphite anode, up to a 3.7 V output voltage can be achieved in the aqueous system. The unique molecular structure of PhSe allows for one Cl^0^ to be stably anchored on poly-PhSe with significantly higher Cl-fixation efficiency compared to other Cl-anchoring strategies. Notably, a desirable three-electron-transfer process (one electron from Cl^0^ to Cl^–^ and two electrons from Se^2+^ to Se^0^) is realized in the fabricated Zn∥poly-PhSe cell, resulting in remarkable electrochemical performance.

## Results and Discussion

### Chlorine Fixation in Organic Se

We explore an organic molecule with the chalcogen group as the Cl-anchoring agent, which is expected to exhibit the superior efficiency of chlorine fixation and cycling stability over the physical adsorption strategy.^22^ 2,1,3-Benzoselenadiazole (denoted as PhSe) is selected as the anchoring agent for ClRR, in which the benzodiazole with strong electron-withdrawing ability can undermine the surface charge density of Se atoms for higher oxidizing ability (Figure 1b), which is favorable for accelerating the conversion reaction kinetics of electrodes.^23^ The active materials are subsequently characterized by Fourier-transform infrared spectroscopy (FTIR) and Raman spectroscopy (Figures S1 and S2), and both the results demonstrate that the organic PhSe molecules are well incorporated in the prepared organic electrodes.

Then, a three-electrode test was conducted to verify the chlorine fixation in the organic Se electrode. The PhSe electrode serves as a working electrode, activated carbon is the counter electrode, and the Ag/AgCl electrode is the reference electrode. The 20 M LiCl electrolyte ensures sufficient Cl^–^ reactant during cycling and suppresses water activity to eliminate oxygen evolution reactions. The working potential range is confined within 0 to 1.3 V to capture the electrochemical features of the ClRR. Subsequently, we collect cyclic voltammetry (CV) curves of the PhSe electrode and AC electrode (Figure 1c). Compared to the AC electrode with weak redox peaks, the PhSe electrode exhibits pronounced cathodic peaks (located at 0.33 and 0.85 V vs Ag/AgCl), indicating high redox activity of the PhSe electrodes.^24,25^ Then *ex situ* X-ray photoelectron spectroscopy (XPS) is employed to examine the redox process of the organic cathodes at different charging states (Figure S3). It is obvious that the two redox peaks in the CV test represent the reversible conversion of (PhSe)^0/2+^ and the Cl^–1/0^, confirming the efficient chlorine fixation in the organic Se electrode.^26,27^ Such a result is further confirmed by the galvanostatic charge–discharge (GCD) curves in Figure 1d, in which a long and flat ClRR plateau (∼1.87 V) is detected for the PhSe electrode with a discharge capacity up to 402 mAh g^–1^ (calculated based on the PhSe mass). The capacity contribution percentages from the redox process of (PhSe)^0/2+^ and Cl^–1/0^ are 54 and 23%, respectively (Figure S4). Normally, the reversibility of cells with ClRR is poor, with extremely low Coulombic efficiency (CE) (∼20%) due to meager Cl fixation in conventional electrodes like AC electrodes.^28^ Notably, the PhSe electrode-based cell with ClRR can achieve a CE up to 92.8% even in the initial charging/discharge process, implying dramatically promoted reversibility of such a PhSe electrode. For comparison, the AC electrode shows a lower CE of 16.3%, further signifying the superiority of organic Se in chlorine fixation to conventional electrodes.

To confirm the Cl-fixation efficiency of the PhSe electrodes, *in situ* gaseous Cl_2_ analysis is adopted to monitor Cl_2_ evolution during the charging process (charged at a constant voltage of 1.2 V for 2 h) (Figure S5). As shown in Figure 1e, at the 0.5 h mark, the Cl_2_ concentration for the PhSe electrode is 0.08 ppm, which is more than an order of magnitude lower than that of the AC electrode (1.1 ppm). During the entire 2 h charging process, the AC electrode shows Cl_2_ above 40 ppm at 1.2 V, whereas the PhSe electrode shows only a small amount (13 ppm at 1.2 V). Clearly, the PhSe electrode can effectively capture oxidized Cl^0^ and significantly reduce the generation of gaseous Cl_2_, thereby promoting the reversibility of batteries with ClRR. Furthermore, the long-term cycling stability of the two electrodes is investigated by the GCD test at 0.5 A g^–1^ (Figure 1f). The reversible capacity of the PhSe electrode remains at 327 mAh g^–1^ after 50 cycles with a high CE above 98.9%. Despite the superiority of the organic Se-based electrode over the AC electrode in the discharging process with ClRR, the PhSe electrode suffers from severe capacity decay (capacity retention 60.2% after 50 cycles), possibly due to the dissolution of organic active materials in electrolytes.^27,29^ Thus, based on the proof-of-concept model of PhSe, it is necessary to improve the structural stability of the organic Se materials to achieve the CIRR with high reversibility and stability.

### High-Voltage Aqueous Batteries

To improve the structural stability of the organic Se electrode, we conducted the copolymerization of 5-ethenyl-2,1,3-benzoselenadiazole and polypyrrole (PPy) (Figure 2a). Introducing PPy aims to improve the electrical conductivity of the polymer electrode for fast conversion kinetics. The as-prepared active materials (denoted as poly-PhSe) exhibit the property of a black powder. The chemical structure of the active material was characterized by FTIR (Figure S6). The peak of C=C within the 5-ethenyl-2,1,3-benzoselenadiazole monomer almost disappeared after the reaction, indicating the successful polymerization of the poly-PhSe.^30^ We also adopted elemental analysis to investigate the content of the element component (Table S1), and the content of the Se atom in the as-synthesized polymer is ∼30.7%.

**Figure 2:**
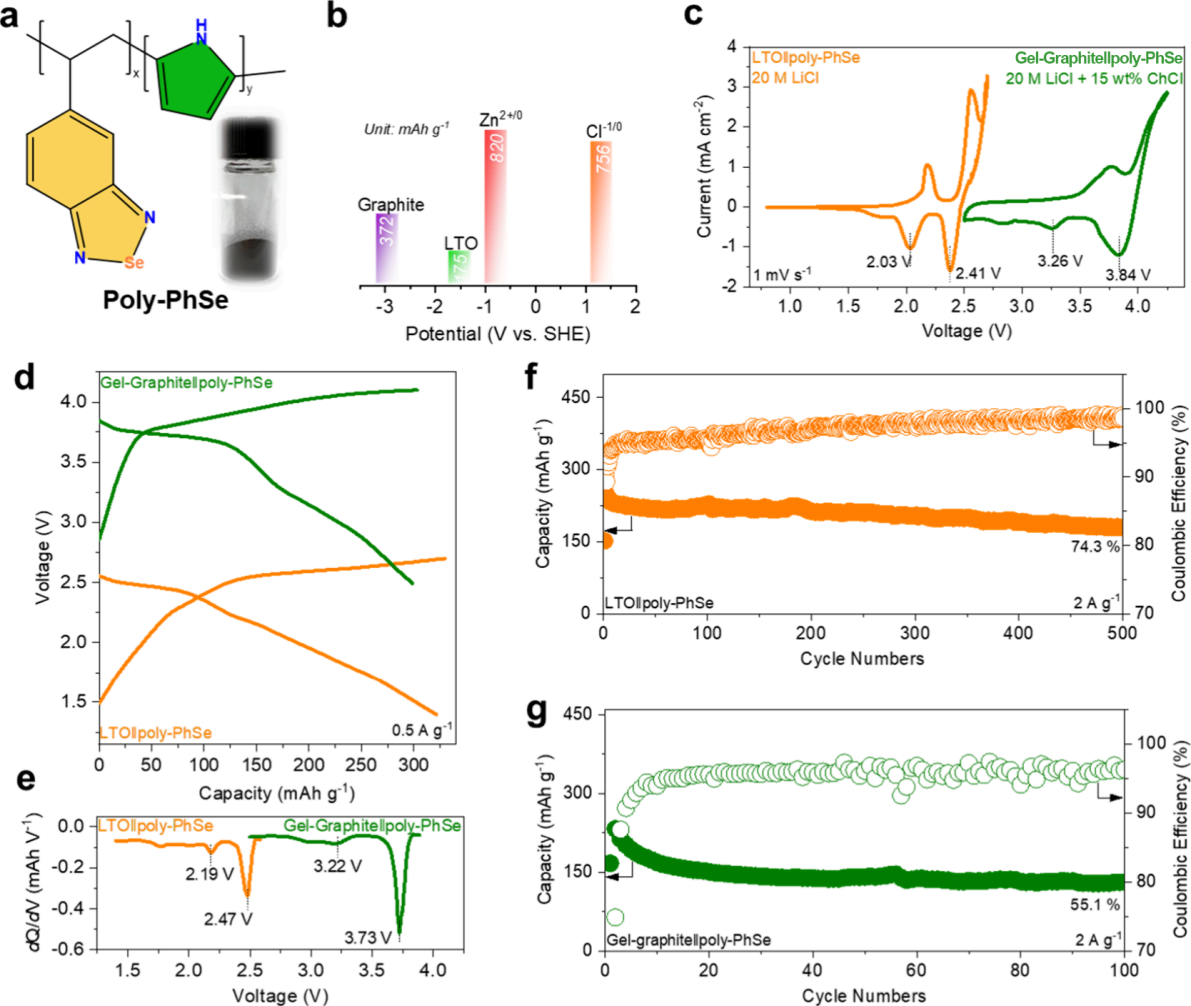
High-voltage aqueous batteries with polymeric organic Se. (a) Schematic illustration of the as-synthesized poly-PhSe and the corresponding optical picture (inset picture); (b) redox potential and the theoretical capacity of the selected electrodes; (c) CV curves and (d) GCD profiles of the LTO∥poly-PhSe cell and graphite∥poly-PhSe cell; (e) dQ/dV vs capacity plot of discharge profiles of the LTO∥poly-PhSe cell and graphite∥poly-PhSe cell at 0.5 A g^–1^. Long-term stability of (f) the LTO∥poly-PhSe cell and (g) graphite∥poly-PhSe cell at 2 A g^–1^.

Then, we screened appropriate anodes for constructing the aqueous poly-PhSe-based battery with CIRR. The characterizations of PhSe and poly-PhSe were independently conducted. Three typical anodes, namely, Zn metal, Li_4_Ti_5_O_12_ (LTO), and graphite anodes, were selected due to their suitable redox potential (Figure 2b). To achieve the reversible cycling of the graphite anode in the challenging aqueous environment, a gel protection layer was cast on the anode, and the detailed fabrication process can be found in the experimental section of the Supporting Information.^4^ We paired the LTO anode and graphite anode with the poly-PhSe cathode and collected the CV curves of the obtained LTO∥poly-PhSe cell and graphite∥poly-PhSe cell (Figure 2c). Two obvious cathodic peaks, located at 2.03 and 2.41 V, can be detected in the CV curves of LTO∥poly-PhSe battery, which can be ascribed to the redox reaction of Se and Cl. When adopting a graphite electrode as the anode, the redox potential of the two redox reactions can be increased to 3.26 and 3.84 V, respectively. Such high-voltage aqueous batteries are rarely reported, indicating the superiority of the as-developed poly-PhSe electrode to other traditional electrodes. The subsequent GCD curves of the two batteries further confirm the high-voltage performance (Figure 2d), in which obvious discharging plateaus located at 2.47 and 2.19 V for the LTO∥poly-PhSe cell and 3.73 and 3.22 V for the graphite∥poly-PhSe cell can be identified (Figure 2e). Up to 323 mAh g^–1^ and 298 mAh g^–1^ discharging capacities can be achieved in LTO∥poly-PhSe cells and graphite∥poly-PhSe cell, respectively. The calculated energy density (based on the mass of active materials) of the poly-PhSe electrode in the two cells can reach 1118 Wh kg^1–^ (graphite∥poly-PhSe cell) and 628 Wh kg^1–^ (LTO∥poly-PhSe cell), respectively. Furthermore, we investigated the long-term cycling performance of the two batteries, which are shown in Figure 2f,g. For the LTO∥poly-PhSe cell, a discharging capacity of around 177 mAh g^–1^ can be maintained after 500 cycles with 74.3% capacity retention. The CE of the battery gradually increased and finally maintained at 98.5%, indicating that highly efficient CIRR can be achieved in the system. For graphite∥poly-PhSe cell, fast capacity decay with low-capacity retention (55.1%) after 100 cycles can be achieved, which can be attributed to the inferior stability and reversibility of graphite anode in the aqueous environment. Generally, up to 3.73 V output voltage is achieved in aqueous batteries with CIRR, which breaks the voltage limit of conventional aqueous batteries.

### Interacting Manners between Poly-PhSe and Cl

To examine the redox process of the Poly-PhSe cathode in LTO∥poly-PhSe cell, we first employ *ex situ* XPS at different charging states (initial, 2.4 and 2.7 V) (Figure 3a,b). At the initial state, two characteristic peaks located at 56.7 and 55.8 eV can be detected, which can be ascribed to the existence of (poly-PhSe)^0^ state. Subsequently, when the battery was charged to 2.4 V, a new peak of Se 3d was detected at 58.8 eV, indicating conversion from (poly-PhSe)^0^ to (poly-PhSe)^2+^ (Figure 3a). With the charging process proceeding (charged to 2.7 V), the oxidative characteristic peak shifts to 59.4 eV, possibly due to bond formation between the positively charged Se and Cl. In addition, we collected XPS spectra of the N 1s core level on the poly-PhSe electrode during cycling (Figure S6). The characteristic N 1s peaks remain unchanged, with a prominent C=N peak and an N–Se peak, indicating that nitrogen atoms do not participate in chlorine fixation. Furthermore, we also investigated the XPS spectra of Cl 2p further to verify the interacting manners between poly-PhSe and Cl. As shown in Figure 3b, the doublet Cl 2p peaks shift to 202.3 and 200.5 eV at charging states 2.6 and 2.7 V, respectively, distinct from the Cl 2p doublet peaks (200.1 and 198.9 eV) of the ionic-state Cl^–^ detected from 2.4 V charging state, indicating the oxidation of Cl^–^ and generation of organic Cl^0^.^31,32^ Considering both the peak shift of Se 2p spectra and the appearance of organic Cl^0^ at charging state 2.7 V, we speculate that (poly-PhSe)^2+^ could bind with Cl^0^ based on the formation of covalent bond and realize Cl fixation at the atomic level. In addition, we also performed *ex situ* XPS analysis on the Se 3d core level of the poly-PhSe electrode in the graphite∥poly-PhSe cell (Figure S7). The results clearly show the characteristic peaks corresponding to both (poly-PhSe)^2+^ and Se–Cl species, confirming that the same conversion process takes place in the graphite∥poly-PhSe cell.

**Figure 3:**
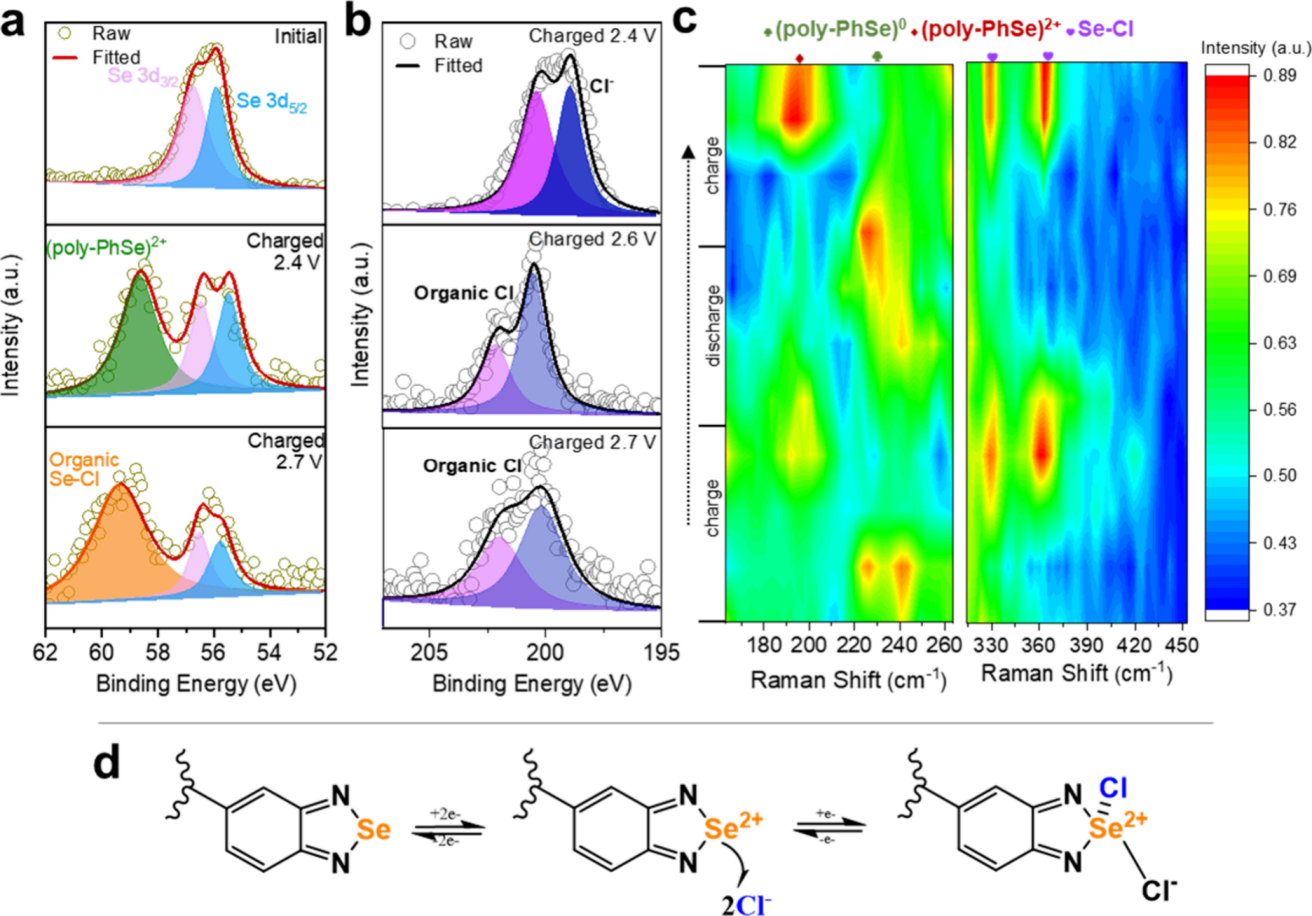
Conversion process and Cl-anchoring mechanism on poly-PhSe electrode. *Ex situ* XPS spectra of (a) Se 3d and (b) Cl 2p at different charging/discharging states; (c) *in situ* Raman patterns at selected potentials; (d) schematic representation for molecular structure transformation during charging.

We further carried out *in situ* Raman characterization to unveil the products during the redox process (Figure 3c). During the charging process, the peak at 226 cm^–1^ gradually disappears, and a new peak appears at 196 cm^–1^, which could be attributed to the conversion from (poly-PhSe)^0^ to (poly-PhSe)^2+^. When the cell was further charged, two new peaks located at 329 and 361 cm^–1^ appeared, which can be ascribed to the formation of organic Se–Cl. When the cell is discharged, the disappearance of organic Se–Cl and (poly-PhSe)^2+^ and the reappearance of (poly-PhSe)^0^ reveal the reversible conversion from (poly-PhSe)^2+^ back to (poly-PhSe)^0^. When the cell is charged again, the (poly-PhSe)^0^ → (poly-PhSe)^2+^ → Se–Cl conversion can be verified by the evolution of the characteristic peaks, revealing the high reversibility of the poly-PhSe-based cell with the ClRR.

Based on the analysis, this conversion reaction accompanied by the corresponding molecular structure transformation is schematically illustrated in Figure 3d, and the details are shown below:

$$\mathrm{Cathode}:\;\mathrm{First}\;\mathrm{step}:\;{\left(\mathrm{poly}-\mathrm{PhSe}\right)}^{0}↔{\left(\mathrm{poly}-\mathrm{PhSe}\right)}^{2+}+2e^{-}\;\mathrm{Third}\;\mathrm{step}:{\left(\mathrm{poly}-\mathrm{PhSe}\right)}^{2+}+2{\mathrm{Cl}}^{-}↔{\left(\mathrm{poly}-{\mathrm{PhSeCl}}_{2}\right)}^{+}+e^{-}$$

### Zn∥PPh-Se Battery with ClRR

Furthermore, we fabricated the Zn∥poly-PhSe cell with a 30 m ZnCl_2_ electrolyte to verify the advancement of the poly-PhSe electrode in zinc batteries. As shown in Figure 4a, the CV curves of this battery show similar redox behaviors compared with the LTO∥poly-PhSe cell and graphite∥poly-PhSe cell, and two cathodic peaks located at 1.66 and 1.12 V can be detected, which are ascribed to the conversion reactions of organic Se–Cl to (poly-PhSe)^2+^ and (poly-PhSe)^2+^ to (poly-PhSe)^0^. In addition, no apparent changes occur in the CV curves even after 15 cycles, indicating this battery’s excellent stability. Then, we collected the GCD curve of the Zn∥poly-PhSe cell, and the Zn∥PPy cell was also fabricated and tested for comparison (Figure 4b). There are no prominent discharging plateaus detected in the Zn∥PPy cell. By contrast, two apparent discharging plateaus located at 1.84 and 1.39 V in the Zn∥poly-PhSe cell can be identified, which could be attributed to the Cl^0^ → Cl^–^ conversion and (poly-PhSe)^2+^ → (poly-PhSe)^0^ conversion, respectively. The whole discharging capacity is around 344 mAh g^–1^ with up to 99% CE. Based on the proposed reaction mechanism, we assume that the discharge curve below 1.45 V could be ascribed to the (poly-PhSe)^2+^ → (poly-PhSe)^0^ conversion. The capacities for the two conversion processes in the discharge curve are 219 mAh g^–1^ (Cl^0/‑^) and 125 mAh g^–1^ ((poly-PhSe)^2+/0^), respectively. Thus, the capacity contribution of each of the two conversion processes is calculated to be 63.7% (Cl^0/‑^) and 36.3% ((poly-PhSe)^2+/0^), respectively. According to the theoretical capacity of Cl (756 mAh g^–1^) and poly-PhSe (96 mAh g^–1^) (based on a one-electron conversion reaction), we further prove that only one Cl^0^ atom is converted to Cl^–^ with a one-electron conversion process, accompanied by a two-electron conversion process corresponding to the reduction reactions of a single poly-PhSe, in accordance with the proposed reaction pathways.

**Figure 4:**
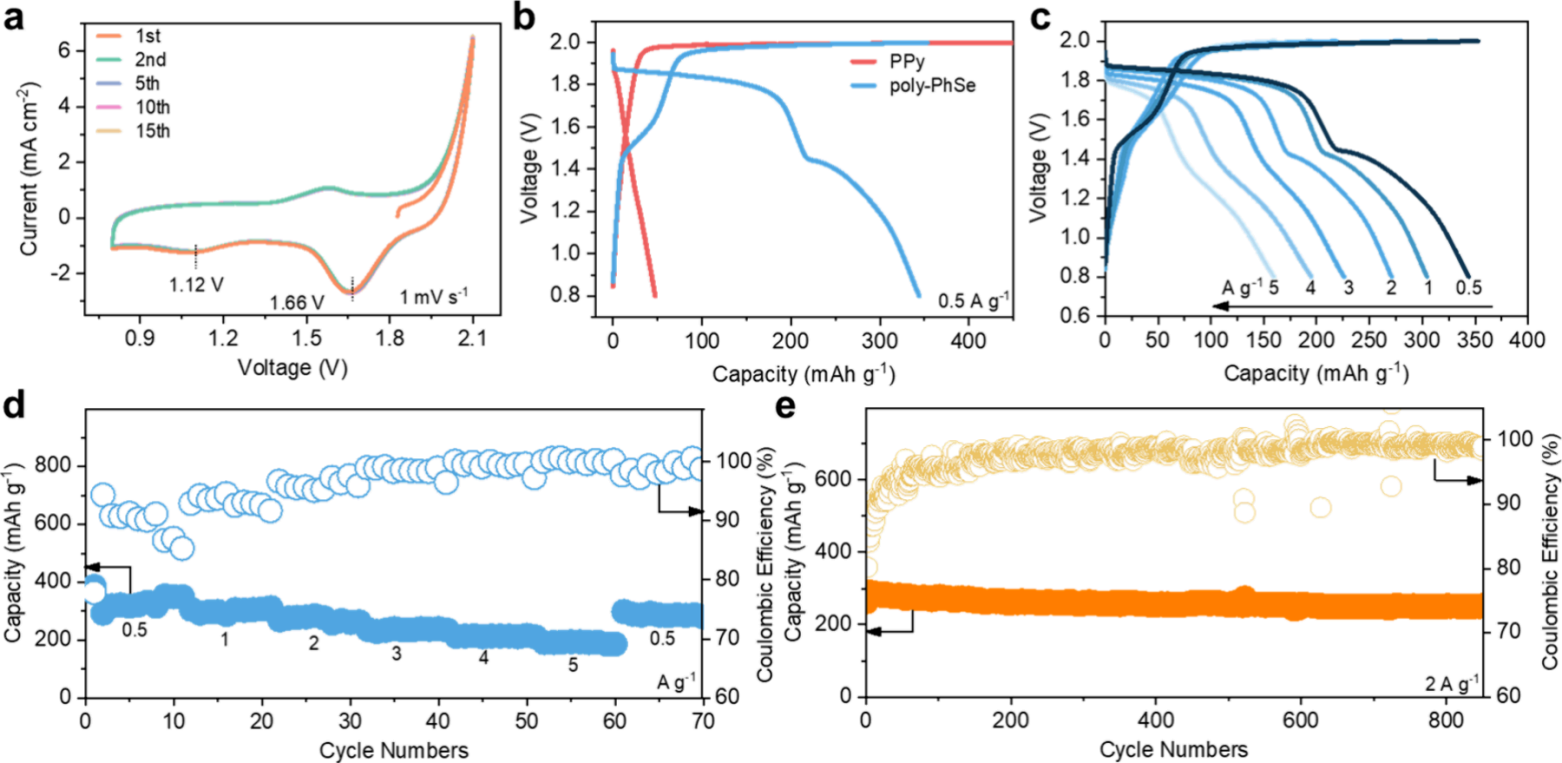
Electrochemical performance of the Zn∥poly-PhSe battery with ClRR. (a) CV curves with different cycles at the scan rate of 1 mV s^–1^; (b) GCD curves at 0.5 A g^–1^; (c) GCD curves at different current densities and (d) the corresponding rate performance; (e) cycling performance at 2 A g^–1^.

Furthermore, the rate performance of the Zn∥poly-PhSe battery is assessed by the GCD curves at different current densities (Figure 4c). The reaction plateaus remain the same at varying current densities, with discharge capacities of 344, 305, 271, 228, 195, and 160 mAh g^–1^ recorded at current densities of 0.5, 1, 2, 3, 4, and 5 A g^–1^, respectively. Specifically, as shown in Figure 4d, as the current density gradually decreases to 0.5 A g^–1^, the capacity almost returns to the original values. In addition, the slight fluctuation of the Coulombic efficiency may be attributed to the irreversible side reactions at the Zn anode side, like corrosion and passivation due to the aqueous ZnCl_2_ electrolyte.^33^ The excellent rate performance of poly-PhSe could be ascribed to the exceedingly good reversibility of the conversion reactions, demonstrating the high tolerance of the poly-PhSe cathode to high-speed conversion reactions.^34^ We compare the rate capability and power density of our Zn∥poly-PhSe battery with that of other reported ZIBs in Figures S8 and S9,^35–47^ and the obviously superior rate performance and power density of our battery confirm the fast conversion kinetics and excellent power output of the poly-PhSe electrode with ClRR. In addition, the capacity contribution from Cl and Se under different current densities is calculated and plotted in Figure S8. With increasing current density, the capacity contribution from Cl and Se remains almost stable, suggesting highly efficient Cl fixation in the poly-PhSe electrode and the fast reaction kinetics of such an organic electrode structure. To evaluate the long-term stability of such a Zn∥poly-PhSe battery, we collect the long-term cycling performance at 2 A g^–1^, as shown in Figure 4e. The reversible capacity retains a value of 252 mAh g^–1^ after 850 cycles with a capacity retention as high as 84.6%, indicating the obviously improved cycling stability with the incorporation of a Zn metal anode. The observed capacity fade can be attributed to the gradual dissolution of organic active materials despite polymerization and to inevitable side reactions at the anode, such as corrosion, passivation, and dendrite formation, which warrant further investigation.^48,49^

### Stability and Practicability of Zn∥poly-PhSe Battery

First, we compared the CE and cycling life of our battery with other reported Cl-based batteries including nonaqueous and aqueous systems.^4,7,11,12,28^ Our Zn∥poly-PhSe battery exhibits significantly improved cycling stability, as evidenced by the high CE (>99.0%) and long cycling life in Figure 5a and Table S2, indicating the superior chlorine fixation performance of the as-prepared poly-PhSe electrode.^9^ Then, benefiting from the promoted ClRR by the poly-PhSe electrode and the resulting flat discharge plateaus, this Zn∥poly-PhSe battery outputs a significantly high-energy density of 682 Wh kg^1–^ (calculated based on the active materials) with an average output voltage of 1.79 V, which is much higher than other reported aqueous batteries (Figure 5b).^35–47^ Furthermore, we also tested the electrochemical performance at varied temperatures (−20 to 50 °C; Figure S9), and a discharge capacity of up to 165 mAh g^–1^ can still be maintained at −20 °C, confirming the excellent low-temperature tolerance of our battery.

**Figure 5:**
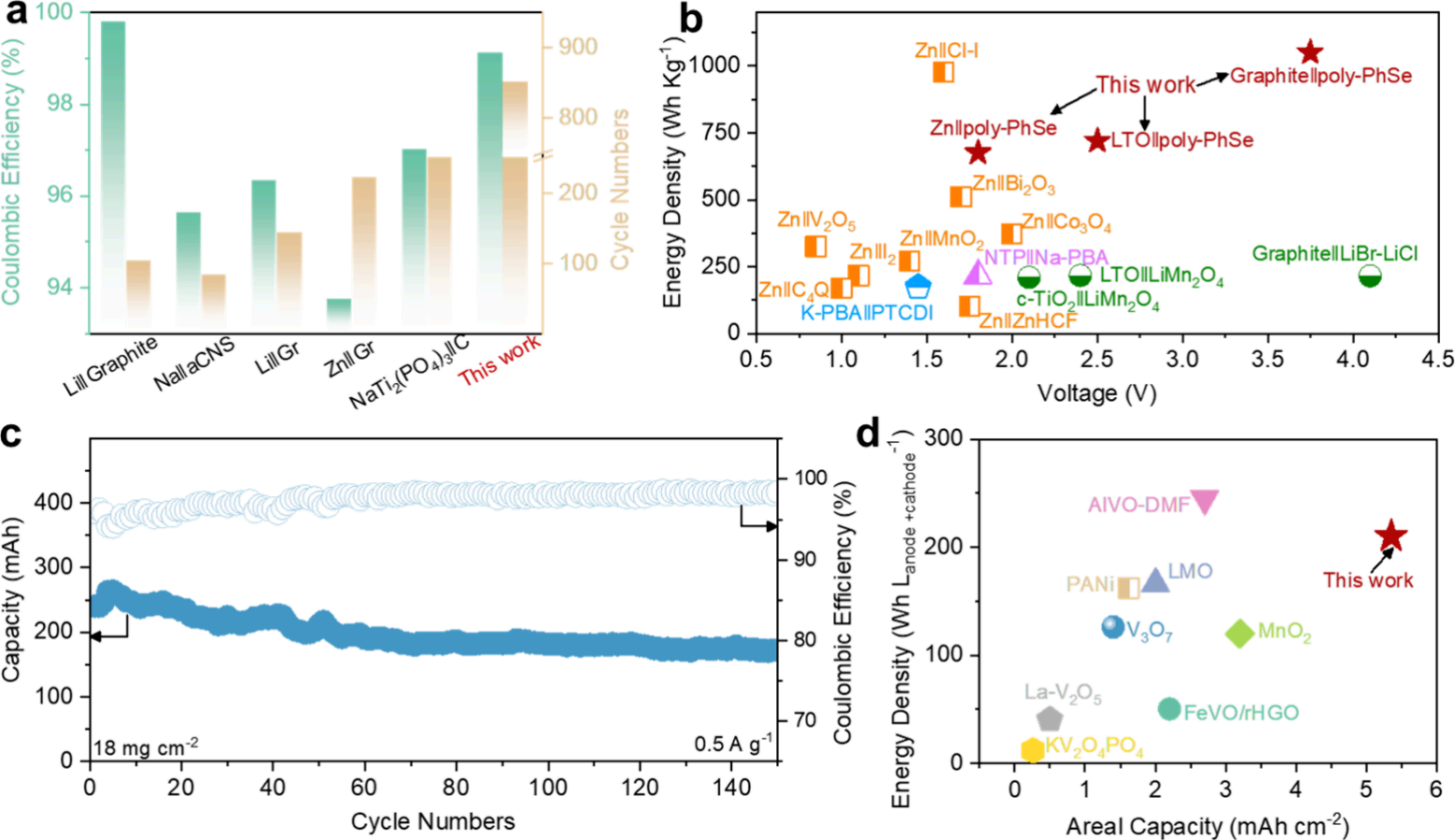
Evaluating the stability and practicability of the Zn∥poly-PhSe battery. (a) Comparison of cycling life and CE with other reports on CIRR,^4,7,11,12,13,28,46^ (b) comparison of energy density and voltage with other reports;^35–47^ (c) cycling performance of the pouch cell with a high loading mass; (d) comparison of areal capacity and voltage of the pouch cell with other reports on aqueous batteries.^41,42,50–55^ C4Q: calix[4]­quinone; PTCDI: perylenetetracarboxylic diimide; PBA: prussian blue analogues; ZnHCF: zinc hexacyanoferrate; PANi: polyaniline; LMO: lithium manganese oxide; rHGO: reduced holey graphene oxide.

Moreover, to assess the potential of our Zn∥poly-PhSe battery for large-scale practical applications, a single-layer pouch cell is fabricated. The pouch cell delivers a discharge capacity of up to 264 mAh at 0.5 A g^–1^ (Figure 5c). Superior cycling stability with a capacity retention of 74.1% (177 mAh after 150 cycles) is achieved in the cell. Additionally, the pouch cell delivers a high areal capacity of 5.3 mAh cm^–2^ (Figure 5d), surpassing all previously reported ZIBs.^41,42,50–55^ In addition, the pouch cell also outputs an energy density of 210 Wh L_cathode+anode_^–1^, indicating the great potential of the developed Zn∥poly-PhSe battery for practical application.^56^ The notable electrochemical performance of the Zn∥poly-PhSe battery suggests the outstanding stability of the poly-PhSe cathode with highly efficient Cl anchoring in this battery. It is worth noting that although the electrochemical performance of the developed Zn∥poly-PhSe battery is promising, the battery cost resulting from the complex selenium-based organic active material and the use of high-concentration electrolytes needs to be further optimized through advanced material development for practical applications.

## Conclusions

Even though ClRR shows excellent potential for constructing high-energy aqueous batteries, the development of ClRR-based batteries is still hindered by poor Cl-fixation efficiency. By exploitation of poly-PhSe as the Cl-anchoring agent, highly efficient Cl fixation at the atomic level has been achieved with one Cl^0^ atom anchored on one single poly-PhSe. The resulting graphite∥poly-PhSe cell exhibits a desired three-electron conversion process, delivering an impressive discharge voltage of 3.7 V. Furthermore, when paired with the Zn anode, a 1.79 V average output voltage and high-discharge capacity (344 mAh g^–1^) can be achieved. Additionally, owing to the stable molecular structures and electrolytes, extraordinary cycling performance is achieved, with a capacity retention as high as 84.6% after 850 cycles. Moreover, the Zn∥poly-PhSe battery also demonstrates remarkable rate performance and a high areal capacity. This design strategy using chalcogen-based anchoring agents opens a new avenue for reversible halogen redox chemistry in batteries.

## Supplementary Material



## Notes

Additional experimental data such as Raman patterns of electrode materials; *ex situ* XPS spectra of electrodes under different charge/discharge states; rate performance and Ragone plot of batteries (PDF)

## References

[1] Liang Y., Yao Y. (2023). Designing modern aqueous batteries. Nat. Rev.
Mater..

[2] Chao D., Zhou W., Xie F., Ye C., Li H., Jaroniec M., Qiao S. Z. (2020). Roadmap
for advanced aqueous batteries:
From design of materials to applications. Sci.
Adv..

[3] Beck F., Rüetschi P. (2000). Rechargeable batteries with aqueous
electrolytes. Electrochim. Acta.

[4] Yang C., Chen J., Ji X., Pollard T. P., Lü X., Sun C.-J., Hou S., Liu Q., Liu C., Qing T., Wang Y., Borodin O., Ren Y., Xu K., Wang C. (2019). Aqueous Li-ion battery enabled by
halogen conversion–intercalation
chemistry in graphite. Nature.

[5] Geng Y., Pan L., Peng Z., Sun Z., Lin H., Mao C., Wang L., Dai L., Liu H., Pan K., Wu X., Zhang Q., He Z. (2022). Electrolyte
additive engineering
for aqueous Zn ion batteries. Energy Storage
Mater..

[6] Zhu Y.-h., Cui Y.-f., Xie Z.-l., Zhuang Z.-b., Huang G., Zhang X.-b. (2022). Decoupled aqueous
batteries using pH-decoupling electrolytes. Nat. Rev. Chem..

[7] Zhu G., Tian X., Tai H.-C., Li Y.-Y., Li J., Sun H., Liang P., Angell M., Huang C.-L., Ku C.-S., Hung W.-H., Jiang S.-K., Meng Y., Chen H., Lin M.-C., Hwang B.-J., Dai H. (2021). Rechargeable Na/Cl2
and Li/Cl2 batteries. Nature.

[8] Jameson A., Gyenge E. (2020). Halogens as Positive
Electrode Active Species for Flow
Batteries and Regenerative Fuel Cells. Electrochem.
Energy Rev..

[9] Kim J. T., Jorné J. (1977). The Kinetics
of a Chlorine Graphite Electrode in the
Zinc-Chlorine Battery. J. Electrochem. Soc..

[10] Kralik D., Jorne J. (1980). Hydrogen Evolution
and Zinc Nodular Growth in the Zinc Chloride Battery. J. Electrochem. Soc..

[11] Zhu G., Liang P., Huang C.-L., Huang C.-C., Li Y.-Y., Wu S.-C., Li J., Wang F., Tian X., Huang W.-H., Jiang S.-K., Hung W.-H., Chen H., Lin M.-C., Hwang B.-J., Dai H. (2022). High-Capacity Rechargeable
Li/Cl2 Batteries with Graphite Positive Electrodes. J. Am. Chem. Soc..

[12] Liu H., Chen C., Yang H., Wang Y., Zou L., Wei Y., Jiang J., Guo J., Shi W., Xu Q., Cheng P. (2020). A Zinc–Dual-Halogen Battery with a Molten Hydrate Electrolyte. Adv. Mater..

[13] Guo Q., Kim K.-I., Li S., Scida A. M., Yu P., Sandstrom S. K., Zhang L., Sun S., Jiang H., Ni Q., Yu D., Lerner M. M., Xia H., Ji X. (2021). Reversible
Insertion of I–Cl Interhalogen in a Graphite Cathode for Aqueous
Dual-Ion Batteries. ACS Energy Lett..

[14] Holleck G. L. (1972). The Reduction
of Chlorine on Carbon in AlCl3 - KCl - NaCl Melts. J. Electrochem. Soc..

[15] Udachin K.
A., Alavi S., Ripmeester J. A. (2013). Water–Halogen Interactions
in Chlorine and Bromine Clathrate Hydrates: An Example of Multidirectional
Halogen Bonding. J. Phys. Chem. C.

[16] Küpper F. C., Feiters M. C., Olofsson B., Kaiho T., Yanagida S., Zimmermann M. B., Carpenter L. J., Luther G. W., Lu Z., Jonsson M., Kloo L. (2011). Commemorating Two Centuries of Iodine
Research: An Interdisciplinary Overview of Current Research. Angew. Chem., Int. Ed..

[17] Lantelme F., Alexopoulos H., Devilliers D., Chemla M. (1991). A Gas Electrode: Behavior
of the Chlorine Injection Electrode in Fused Alkali Chlorides. J. Electrochem. Soc..

[18] Kim K.-i., Guo Q., Tang L., Zhu L., Pan C., Chang C.-h., Razink J., Lerner M. M., Fang C., Ji X. (2020). Reversible
Insertion of Mg-Cl Superhalides in Graphite as a Cathode for Aqueous
Dual-Ion Batteries. Angew. Chem., Int. Ed..

[19] Skinner H. (1945). A revision
of some bond-energy values and the variation of bond-energy with bond-length. Trans. Faraday Soc..

[20] Krebs, B. ; Ahlers, F.-P. , Developments in chalcogen-halide chemistry. In Adv. Inorg. Chem.; Elsevier: 1990; Vol. 35, pp 235–317.

[21] Chen Z., Hou Y., Wang Y., Wei Z., Chen A., Li P., Huang Z., Li N., Zhi C. (2024). Selenium-Anchored Chlorine
Redox Chemistry in Aqueous Zinc Dual-Ion Batteries. Adv. Mater..

[22] Huang J., Dong X., Guo Z., Wang Y. (2020). Progress of organic
electrodes in aqueous electrolyte for energy storage and conversion. Angew. Chem..

[23] Cui H., Wang T., Huang Z., Liang G., Chen Z., Chen A., Wang D., Yang Q., Hong H., Fan J., Zhi C. (2022). High-Voltage Organic Cathodes for Zinc-Ion Batteries
through Electron Cloud and Solvation Structure Regulation. Angew. Chem..

[24] Chen Z., Cui H., Hou Y., Wang X., Jin X., Chen A., Yang Q., Wang D., Huang Z., Zhi C. (2022). Anion chemistry
enabled positive valence conversion to achieve a record high-voltage
organic cathode for zinc batteries. Chem..

[25] Chen Z., Yang Q., Mo F., Li N., Liang G., Li X., Huang Z., Wang D., Huang W., Fan J., Zhi C. (2020). Aqueous Zinc–Tellurium
Batteries with Ultraflat Discharge
Plateau and High Volumetric Capacity. Adv. Mater..

[26] Zhang X. F., Jiao S. Q., Tu J., Song W. L., Xiao X., Li S., Wang M. Y., Lei H. P., Tian D. H., Chen H. S., Fang D. N. (2019). Rechargeable ultrahigh-capacity tellurium–aluminum
batteries. Energy Environ. Sci..

[27] Chen Z., Mo F., Wang T., Yang Q., Huang Z., Wang D., Liang G., Chen A., Li Q., Guo Y., Li X., Fan J., Zhi C. (2021). Zinc/selenium conversion battery:
a system highly compatible with both organic and aqueous electrolytes. Energy Environ. Sci..

[28] Hou S., Chen L., Fan X., Fan X., Ji X., Wang B., Cui C., Chen J., Yang C., Wang W., Li C., Wang C. (2022). High-energy and low-cost
membrane-free chlorine flow battery. Nat. Commun..

[29] Huang X., Liu Y., Liu C., Zhang J., Noonan O., Yu C. (2018). Rechargeable
aluminum–selenium batteries with high capacity. Chem. Sci..

[30] Masruroh, Santjojo D. J. D. H., Abdurrouf, Abdillah M. A., Padaga M. C., Sakti S. P. (2019). Effect of Electron Density and Temperature
in Oxygen Plasma Treatment of Polystyrene Surface. IOP Conf. Ser.: Mater. Sci. Eng..

[31] Lannon J. M., Meng Q. (1999). Analysis of
a Filled Poly­(vinyl chloride)
Polymer by XPS. Surf. Sci. Spectra.

[32] Araujo J. R., Archanjo B. S., de Souza K. R., Kwapinski W., Falcão N. P. S., Novotny E. H., Achete C. A. (2014). Selective
extraction
of humic acids from an anthropogenic Amazonian dark earth and from
a chemically oxidized charcoal. Biol. Fertil.
Soils.

[33] Chen Z., Wang S., Wei Z., Wang Y., Wu Z., Hou Y., Zhu J., Wang Y., Liang G., Huang Z., Chen A., Wang D., Zhi C. (2023). Tellurium with Reversible
Six-Electron Transfer Chemistry for High-Performance Zinc Batteries. J. Am. Chem. Soc..

[34] Li X. L., Li M., Huang Z. D., Liang G. J., Chen Z., Yang Q., Huang Q., Zhi C. Y. (2021). Activating the I-0/I+ redox couple
in an aqueous I-2-Zn battery to achieve a high voltage plateau. Energy Environ. Sci..

[35] Liu Z., Yang Q., Wang D., Liang G., Zhu Y., Mo F., Huang Z., Li X., Ma L., Tang T., Lu Z., Zhi C. (2019). A Flexible Solid-State Aqueous Zinc Hybrid Battery
with Flat and High-Voltage Discharge Plateau. Adv. Energy Mater..

[36] Ma L., Chen S., Li H., Ruan Z., Tang Z., Liu Z., Wang Z., Huang Y., Pei Z., Zapien J. A., Zhi C. (2018). Initiating
a mild aqueous electrolyte Co3O4/Zn battery with 2.2 V-high
voltage and 5000-cycle lifespan by a Co­(iii) rich-electrode. Energy Environ. Sci..

[37] Liang G. J., Mo F. N., Wang D. H., Li X. L., Huang Z. D., Li H. F., Zhi C. Y. (2020). Commencing mild Ag–Zn batteries
with long-term stability and ultra-flat voltage platform. Energy Storage Mater..

[38] Wang D., Zhao Y., Liang G., Mo F., Li H., Huang Z., Li X., Tang T., Dong B., Zhi C. (2020). A zinc battery with ultra-flat discharge plateau through phase transition
mechanism. Nano Energy.

[39] Pan H. L., Li B., Mei D. H., Nie Z. M., Shao Y. Y., Li G. S., Li X. S., Han K. S., Mueller K. T., Sprenkle V., Liu J. (2017). Controlling
Solid–Liquid Conversion Reactions for a Highly
Reversible Aqueous Zinc–Iodine Battery. ACS Energy Lett..

[40] Zhang N., Dong Y., Jia M., Bian X., Wang Y., Qiu M., Xu J., Liu Y., Jiao L., Cheng F. (2018). Rechargeable
Aqueous Zn–V2O5 Battery with High Energy Density and Long Cycle
Life. ACS Energy Lett..

[41] Wang D. H., Wang L. F., Liang G. J., Li H. F., Liu Z. X., Tang Z. J., Liang J. B., Zhi C. Y. (2019). A Superior δ-MnO2
Cathode and a Self-Healing Zn-δ-MnO2 Battery. ACS Nano.

[42] Zhao Q., Huang W., Luo Z., Liu L., Lu Y., Li Y., Li L., Hu J., Ma H., Chen J. (2018). High-capacity
aqueous zinc batteries using sustainable quinone electrodes. Sci. Adv..

[43] Ghanbari K., Mousavi M. F., Shamsipur M., Karami H. (2007). Synthesis of polyaniline/graphite
composite as a cathode of Zn-polyaniline rechargeable battery. J. Power Sources.

[44] Yuan G., Bai J., Doan T. N. L., Chen P. (2014). Synthesis and electrochemical investigation
of nanosized LiMn2O4 as cathode material for rechargeable hybrid aqueous
batteries. Mater. Lett..

[45] He P., Quan Y., Xu X., Yan M., Yang W., An Q., He L., Mai L. (2017). High-Performance
Aqueous Zinc–Ion
Battery Based on Layered H2V3O8 Nanowire Cathode. Small.

[46] Zhang L., Chen L., Zhou X., Liu Z. (2015). Towards High-Voltage
Aqueous Metal-Ion Batteries Beyond 1.5 V: The Zinc/Zinc Hexacyanoferrate
System. Adv. Energy Mater..

[47] Li H., Yang Q., Mo F., Liang G., Liu Z., Tang Z., Ma L., Liu J., Shi Z., Zhi C. (2019). MoS2 nanosheets with expanded interlayer
spacing for rechargeable
aqueous Zn-ion batteries. Energy Storage Mater..

[48] Du W., Du X., Ma M., Huang S., Sun X., Xiong L. (2022). Polymer Electrode
Materials for Lithium-Ion Batteries. Adv. Funct.
Mater..

[49] Yang L., Fu Y., Liu H., Nie Q., Zhang M., Shen Z. (2023). Investigating
the Zinc Deposition Behavior in Aqueous Zinc-Ion Batteries with PEG/Cellulose/ZnCl2
Water-In-Salt Electrolytes via a Homemade Visualized Three-Electrode
Tubular Cell. ACS Sustainable Chem. Eng..

[50] Chen D., Lu M., Wang B., Cheng H., Yang H., Cai D., Han W., Fan H. J. (2021). High-mass loading V3O7·H2O nanoarray for Zn-ion
battery: New synthesis and two-stage ion intercalation chemistry. Nano Energy.

[51] Hao Y., Zhou J., Wei G., liu A., Zhang Y., Mei Y., Lu B., Luo M., Xie M. (2021). Artificial N-doped
Graphene Protective Layer Enables Stable Zn Anode for Aqueous Zn-ion
Batteries. ACS Appl. Energy Mater..

[52] Zhang D., Cao J., Yue Y., Pakornchote T., Bovornratanaraks T., Han J., Zhang X., Qin J., Huang Y. (2021). Two Birds with One
Stone: Boosting Zinc-Ion Insertion/Extraction Kinetics and Suppressing
Vanadium Dissolution of V2O5 via La3+ Incorporation Enable Advanced
Zinc-Ion Batteries. ACS Appl. Mater. Interfaces.

[53] Yang X., Deng W., Chen M., Wang Y., Sun C. (2020). Mass-Producible,
Quasi-Zero-Strain, Lattice-Water-Rich Inorganic Open-Frameworks for
Ultrafast-Charging and Long-Cycling Zinc-Ion Batteries. Adv. Mater..

[54] Zhou J., Xie M., Wu F., Mei Y., Hao Y., Li L., Chen R. (2022). Encapsulation of Metallic
Zn in a Hybrid MXene/Graphene Aerogel as
a Stable Zn Anode for Foldable Zn-Ion Batteries. Adv. Mater..

[55] Ma H., Tian X., Wang T., Tang K., Liu Z., Hou S., Jin H., Cao G. (2021). Tailoring Pore Structures of 3D Printed
Cellular High-Loading Cathodes for Advanced Rechargeable Zinc-Ion
Batteries. Small.

[56] Yang Q., Li X., Chen Z., Huang Z., Zhi C. (2022). Cathode Engineering
for High Energy Density Aqueous Zn Batteries. Acc. Mater. Res..

