# Supplementary material for: Highly Efficient Chlorine Fixation Based on Organic Selenium for 3.7‑V Aqueous Batteries
Source: J Am Chem Soc. 2026 Mar 16;148(11):11531–9. doi: 10.1021/jacs.5c16178 (PMC13081107; doi:10.1021/jacs.5c16178)
Supplement: Supplementary file 1 [file ja5c16178_si_001.pdf]

Supporting Information

Highly Efficient Chlorine Fixation based on Organic Selenium for 3.7-V Aqueous Batteries

Ze Chen*[1], Yiqiao Wang[2], Zhiquan Wei[2], Ao Chen[2], Xinliang Li*[3], Zhaodong Huang[4], Shixun Wang,[5] Chunyi Zhi*[2, 5]

[1] Wu Jieh Yee School of Interdisciplinary Studies, Lingnan University, 8 Castle Peak Road, Tuen Mun, Hong Kong 999077, China.

[2] Department of Materials Science and Engineering, City University of Hong Kong, 83 Tat Chee Avenue, Kowloon, Hong Kong 999077, China.

[3] School of Physics and Laboratory of Zhongyuan Light, Zhengzhou University, Zhengzhou 450052, China.

[4] Department of Chemical and Biological Engineering, Hong Kong University of Science and Technology, Clear Water Bay, Kowloon, Hong Kong 999077, China

[5] Department of Mechanical Engineering, The University of Hong Kong, Pokfulam, Hong Kong 999077, China.

E-mail: zechen2@ln.edu.hk; lixinliang@zzu.edu.cn; cyzhi@hku.hk

Experimental Section

Materials: 2,1,3-Benzoselenadiazole (PhSe), Pyrrole, Zinc chloride ($ZnCl_2$), Ammonium persulfate, Choline chloride (ChCl), 1,1,2,2-tetrafluoroethyl-2′,2′,2′-trifluoroethyl ether (HFE), Bis(trifluoromethane)sulfonimide lithium (LiTFSI), Fluoroethylene carbonate, Polyethylene oxide and N-Methyl-2-pyrrolidone (NMP) are purchased from Aladdin. 5-ethenyl-2,1,3-benzoselenadiazole was purchased from Nornachem Co., Ltd. Poly(tetrafluoroethylene) (PTFE) binder, poly(vinylidene fluoride), acetylene blacks, super p and activated carbon (AC), lithium titanate ($Li_4Ti_5O_{12}$) and graphite were purchased from CANRD company. Pyrrole (Aldrich, 99%) is used after distillation under reduced pressure and stored under an argon atmosphere at 4 °C. Other chemicals are analytically pure and used as received without any further purification.

Synthesis of Poly(5-ethenyl-2,1,3-benzoselenadiazole)-*co*-polypyrrole (poly-PhSe): A typical operation procedure of synthesis is given as follows: 50 mL deionized water is added into a three-necked round-bottom flask and deoxygenated for 30 min followed by introducing 10 mmol 5-ethenyl-2,1,3-benzoselenadiazole monomer and 1 mmol pyrrole monomer. After 10 min, 0.15g ammonium persulfate is added to the reaction mixture. The solution is stirred under a nitrogen atmosphere and the polymerization experiments are carried out at room temperature for 0.5 h and 60 ºC for 12 h. After polymerization, the products are washed by several circles of centrifugation with water and alcohol and dried in a vacuum oven at 65 ºC.

Preparation of PhSe and poly-PhSe electrodes: To prepare the PhSe and poly-PhSe cathodes, PhSe/poly-PhSe powders, acetylene blacks, and PTFE binder were mixed based on the mass ratio 7:2:1 to form a homogeneous slurry, which was then cast onto a piece of carbon cloth. The electrode was further dried at 45 °C for 6 hours. The effective loading mass of active materials was controlled

at around 3 mg cm^{-2} and 18 mg cm^{-2} (for the large-scale pouch cell).

Preparation of lithium titanate (LTO) electrode and graphite electrode: LTO or graphite was mixed with super p and PVDF with the ratio of 8:1:1, then NMP was added into the mixture to form a homogeneous slurry. The slurry was cast on the titanium foil, and the electrodes were dried at 80 °C under a vacuum for 8 hours. The effective loading mass of active materials was controlled at around 10 mg cm^{-2} (LTO) and 5 mg cm^{-2} (graphite). A gel protection layer was prepared to coat on the graphite electrode to be applied in the challenging aqueous environment. The preparation process is as follows: the coating gel is prepared by mixing HFE with 0.5 M LiTFSI (denoted as LiTFSI-HFE gel) and 10 wt% PEO in HFE/fluoroethylene carbonate (with volume ratio 95:5) and heated at 70 °C for 5 min under vigorous stirring.

Characterization methods: X-ray photoelectron spectroscopy (XPS, ESCALAB 250) was employed to analyze the variation of surface elements in the electrodes at different states. The electrode was rinsed with plenty of deionized water and then immersed in the deionized water for 30 min and repeated three times. Subsequently, the electrode was dried under vacuum for the test. Raman spectra were recorded with a WITec alpha300 access with a 532 nm laser. The gas concentration detection of gaseous Cl_2 was measured by Kallu Electronics detector based on a home-designed cell and the measuring protocols have been illustrated in Figure S5. Fourier-transform infrared spectroscopy (FTIR) was collected based on the PerkinElmer Spectrum II. The elemental composition of the materials was collected based on the element analyzer ELEMENTRAC.

Electrochemical measurements: In this study, the three-electrode electrochemical test was conducted based on the Swagelok T-cells, where the working electrode (PhSe electrode) and the

counter electrode (AC electrode) are separated by the separator comprising two layers of glass-fiber membranes. The reference electrode is Ag/AgCl electrode. For the common electrochemical test, the two-electrode configuration based on the Swagelok cell was applied, where poly-PhSe serves as the cathode, LTO or graphite or Zn metal foil serves as the anode. 30 m $ZnCl_2$, 20 M LiCl and 20 M LiCl with 15 wt% ChCl serves as the electrolyte of Zn‖poly-PhSe, LTO‖poly-PhSe and graphite‖poly-PhSe, respectively. In addition, pouch cells were also assembled for electrochemical measurements. The pouch cell was assembled using a zinc foil (0.2 mm) as the anode electrode, a poly-PhSe electrode with titanium mesh as the current collector (18 mg cm^{-2} loading mass) as the cathode, and a glass fiber membrane as the separator. The cell assembly was completed by stacking the cathode, separator, and anode in a pouch configuration, after which the 30 m $ZnCl_2$ aqueous electrolyte was injected. The cyclic performance and electrochemical properties of batteries were characterized by the LAND CT2001A device and the electrochemical workstation CHI 760D at 25 °C. For each electrochemical test, at least five cells were assembled to guarantee performance repeatability.

Computational details: The electronic structure calculations, including the geometries, energies, and frequencies of all the stationary points (the reactants, transition states (TSs), and products), were performed by the GAUSSIAN 09 program. Density functional theory calculations (DFT) were carried out by using the B3LYP (Becke's three-parameter nonlocal-exchange functional[1] with the gradient-corrected of Lee, Yang, and Parr[2]) method.

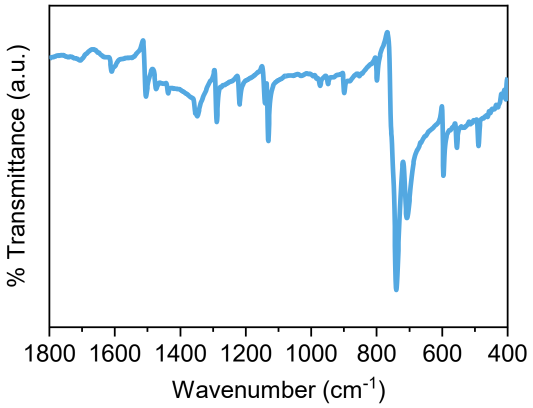

Figure S1. FTIR of the PhSe electrode.

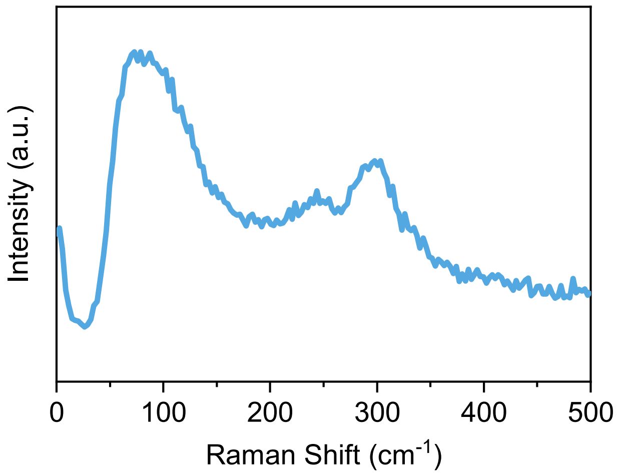

Figure S2. Raman spectra of the PhSe electrode.

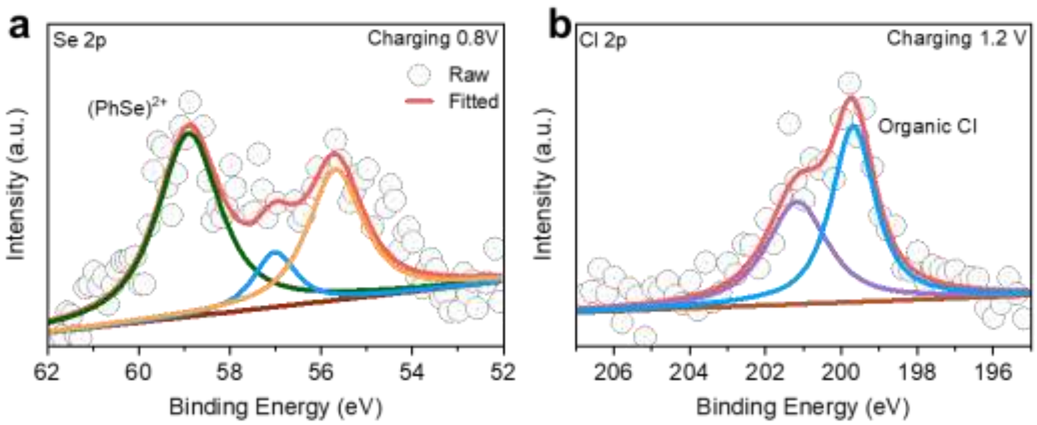

Figure S3. *Ex-situ* XPS spectra of PhSe electrodes at different charging states: a) Se 2p; b) Cl 2p.

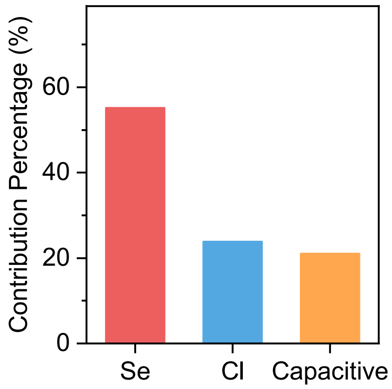

Figure S4. Capacity contribution percentages of the diverse redox process in the PhSe electrode.

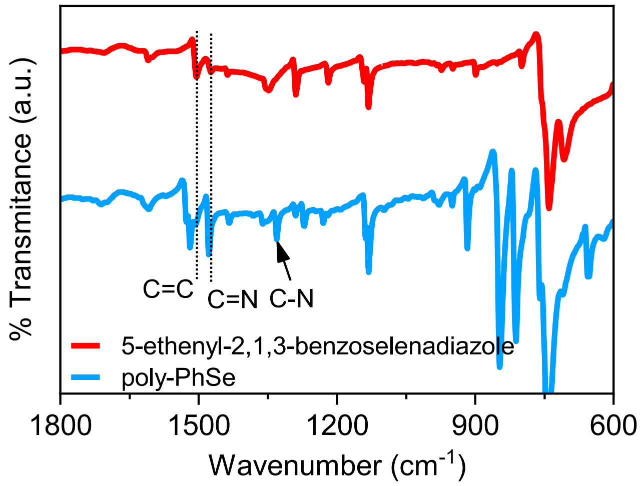

Figure S5. FTIR of the as-synthesized poly-PhSe.

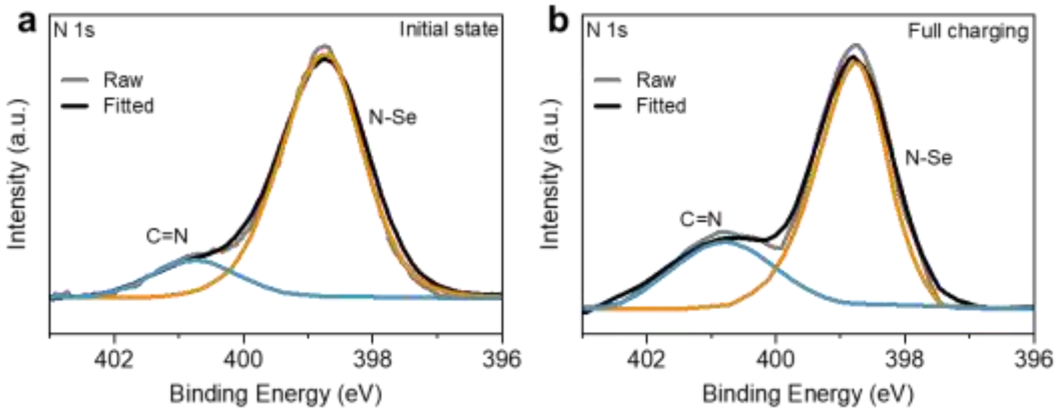

Figure S6. *Ex-situ* XPS spectra on the N 1s core level of the poly-PhSe electrode at different charging states: a) initial state and b) full charging.

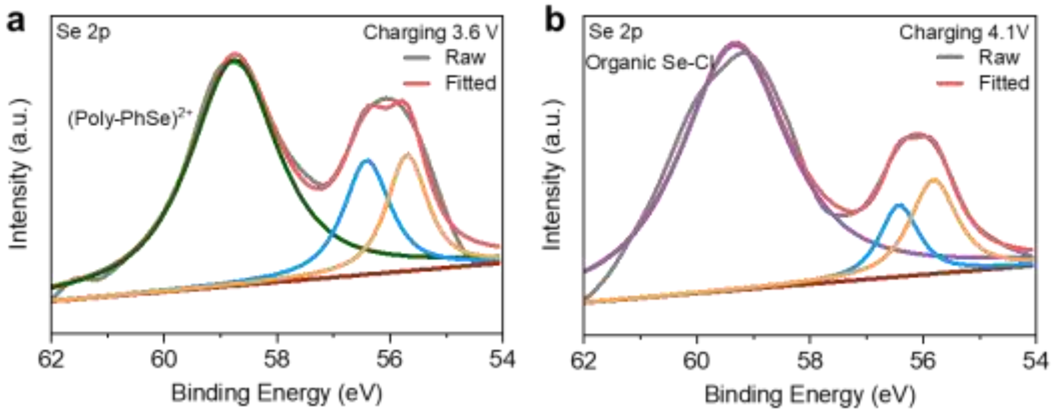

Figure S7. *Ex-situ* XPS spectra on the Se 3d core level of the poly-PhSe electrode in graphite‖poly-PhSe cell at different charging states: a) charging 3.6 V and b) charging 4.1 V.

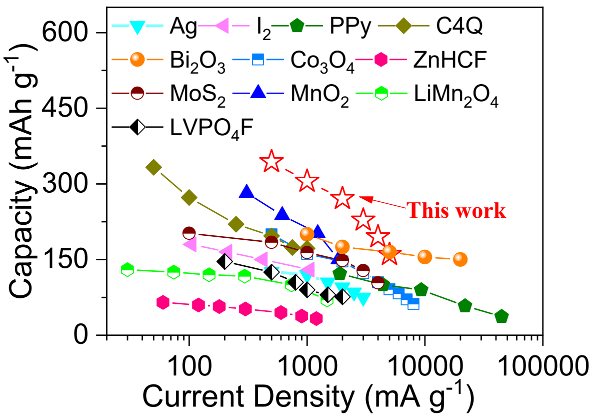

Figure S8. Comparison of the rate performance with other reports.[3-15]

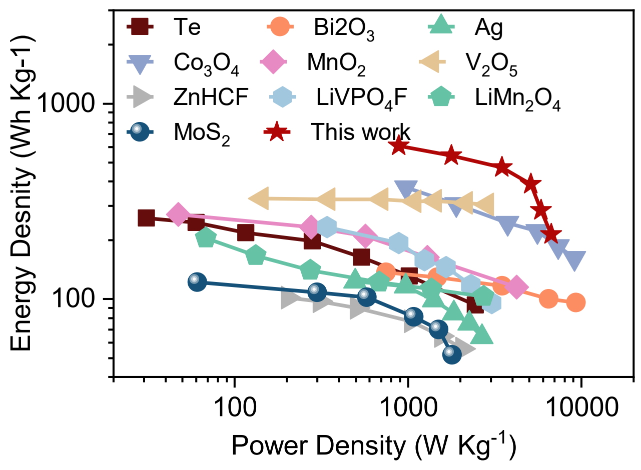

Figure S9. Ragone plot of Zn||poly-PhSe battery with other reports.[3-6, 8, 9, 11-15]

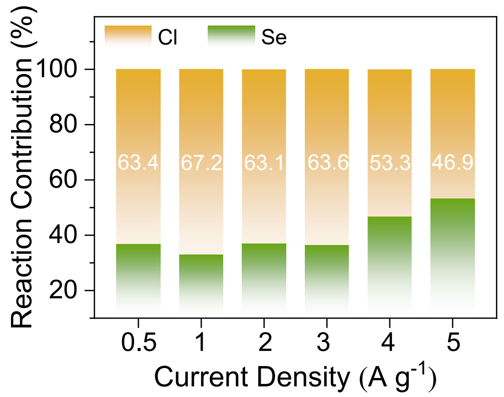

Figure S10. Capacity contribution from Se and Cl at different current densities.

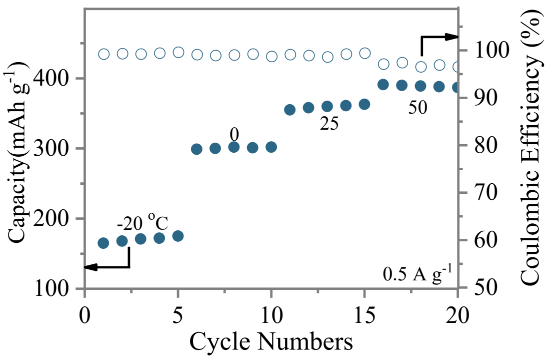

Figure S11. Discharge capacity of the Zn||poly-PhSe battery at varied temperatures.

Table S1. The content of element components in the poly-PhSe active materials.

Element	Percentage (%)
C	53.1
N	16.2
Se	30.7

Table S2. Comparison of our Zn||poly-PhSe batteries with other chlorine batteries.

Battery types	Specific capacities (mAh g^{-1})	Voltage output (V)	Coulombic Efficiency (%)	Cycling performance (%)	Ref
Li||Graphite	243 (0.08 A g^{-1})	4.1	99.8	74% after 150 cycles (0.08 A g^{-1})	[16]
Na||aCNS	2800 (0.05 A g^{-1})	3.2	95.6	91.7% after 80 cycles (0.1 A g^{-1})	[17]
Li||Graphite	375 (0.15 A g^{-1})	3.5	96.3	92.1% after 140 cycles (0.1 A g^{-1})	[18]
Zn||Graphite	260 (0.1 A g^{-1})	1.62	93.7	78% after 100 cycles (0.25 A g^{-1})	[19]
$NaTi_2(PO_4)_3$||C	97 Ah L^{-1} (10 m A cm^{-2})	1.76	97.0	86.7% after 500 cycles (20 mA cm^{-2})	[20]
Zn||poly-PhSe	344 (0.5 A g^{-1})	1.79	99.1	84.6% after 850 cycles (2 A g^{-1})	This work

References

1. Lee, C.; Yang, W.; Parr, R. G. Development of the Colle-Salvetti correlation-energy formula into a functional of the electron density. ***Phys Rev B Condens Matter*** 1988, 37, 785-789.
2. Becke, A. D. A new mixing of Hartree–Fock and local density-functional theories. ***The Journal of chemical physics*** 1993, 98, 1372-1377.
3. Liu, Z. X.; Yang, Q.; Wang, D. H.; Liang, G. J.; Zhu, Y. H.; Mo, F. N.; Huang, Z. D.; Li, X. L.; Ma, L. T.; Tang, T. C.; Lu, Z. G.; Zhi, C. Y. A Flexible Solid-State Aqueous Zinc Hybrid Battery with Flat and High-Voltage Discharge Plateau. ***Adv. Energy Mater.*** 2019, 9, 1902473.
4. Ma, L.; Chen, S.; Li, H.; Ruan, Z.; Tang, Z.; Liu, Z.; Wang, Z.; Huang, Y.; Pei, Z.; Zapien, J. A.; Zhi, C. Initiating a mild aqueous electrolyte Co3O4/Zn battery with 2.2 V-high voltage and 5000-cycle lifespan by a Co(iii) rich-electrode. ***Energy Environ. Sci.*** 2018, 11, 2521-2530.
5. Liang, G. J.; Mo, F. N.; Wang, D. H.; Li, X. L.; Huang, Z. D.; Li, H. F.; Zhi, C. Y. Commencing mild Ag–Zn batteries with long-term stability and ultra-flat voltage platform. ***Energy Storage Mater.*** 2020, 25, 86-92.
6. Wang, D.; Zhao, Y.; Liang, G.; Mo, F.; Li, H.; Huang, Z.; Li, X.; Tang, T.; Dong, B.; Zhi, C. A zinc battery with ultra-flat discharge plateau through phase transition mechanism. ***Nano Energy*** 2020, 71, 104583.
7. Pan, H. L.; Li, B.; Mei, D. H.; Nie, Z. M.; Shao, Y. Y.; Li, G. S.; Li, X. S.; Han, K. S.; Mueller, K. T.; Sprenkle, V.; Liu, J. Controlling Solid–Liquid Conversion Reactions for a Highly Reversible Aqueous Zinc–Iodine Battery. ***ACS Energy Lett.*** 2017, 2, 2674-2680.
8. Zhang, N.; Dong, Y.; Jia, M.; Bian, X.; Wang, Y.; Qiu, M.; Xu, J.; Liu, Y.; Jiao, L.; Cheng, F. Rechargeable Aqueous Zn–V2O5 Battery with High Energy Density and Long Cycle Life. ***ACS Energy Lett.*** 2018, 3, 1366-1372.

9. Wang, D. H.; Wang, L. F.; Liang, G. J.; Li, H. F.; Liu, Z. X.; Tang, Z. J.; Liang, J. B.; Zhi, C. Y. A Superior δ-MnO2 Cathode and a Self-Healing Zn-δ-MnO2 Battery. ***ACS Nano*** 2019, 13, 10643-10652.
10. Zhao, Q.; Huang, W.; Luo, Z.; Liu, L.; Lu, Y.; Li, Y.; Li, L.; Hu, J.; Ma, H.; Chen, J. High-capacity aqueous zinc batteries using sustainable quinone electrodes. ***Sci. Adv.*** 2018, 4, eaao1761.
11. Ghanbari, K.; Mousavi, M. F.; Shamsipur, M.; Karami, H. Synthesis of polyaniline/graphite composite as a cathode of Zn-polyaniline rechargeable battery. ***J. Power Sources*** 2007, 170, 513-519.
12. Yuan, G.; Bai, J.; Doan, T. N. L.; Chen, P. Synthesis and electrochemical investigation of nanosized LiMn2O4 as cathode material for rechargeable hybrid aqueous batteries. ***Mater. Lett.*** 2014, 137, 311-314.
13. He, P.; Quan, Y.; Xu, X.; Yan, M.; Yang, W.; An, Q.; He, L.; Mai, L. High-Performance Aqueous Zinc–Ion Battery Based on Layered H2V3O8 Nanowire Cathode. ***Small*** 2017, 13, 1702551.
14. Zhang, L.; Chen, L.; Zhou, X.; Liu, Z. Towards High-Voltage Aqueous Metal-Ion Batteries Beyond 1.5 V: The Zinc/Zinc Hexacyanoferrate System. ***Adv. Energy Mater.*** 2015, 5, 1400930.
15. Li, H.; Yang, Q.; Mo, F.; Liang, G.; Liu, Z.; Tang, Z.; Ma, L.; Liu, J.; Shi, Z.; Zhi, C. MoS2 nanosheets with expanded interlayer spacing for rechargeable aqueous Zn-ion batteries. ***Energy Storage Mater.*** 2019, 19, 94-101.
16. Yang, C.; Chen, J.; Ji, X.; Pollard, T. P.; Lü, X.; Sun, C.-J.; Hou, S.; Liu, Q.; Liu, C.; Qing, T.; Wang, Y.; Borodin, O.; Ren, Y.; Xu, K.; Wang, C. Aqueous Li-ion battery enabled by halogen conversion–intercalation chemistry in graphite. ***Nature*** 2019, 569, 245-250.
17. Zhu, G.; Tian, X.; Tai, H.-C.; Li, Y.-Y.; Li, J.; Sun, H.; Liang, P.; Angell, M.; Huang, C.-L.; Ku, C.-S.; Hung, W.-H.; Jiang, S.-K.; Meng, Y.; Chen, H.; Lin, M.-C.; Hwang, B.-J.; Dai, H. Rechargeable Na/Cl2 and Li/Cl2 batteries. ***Nature*** 2021, 596, 525-530.
18. Zhu, G.; Liang, P.; Huang, C.-L.; Huang, C.-C.; Li, Y.-Y.; Wu, S.-C.; Li, J.; Wang, F.; Tian, X.; Huang, W.-H.; Jiang, S.-K.; Hung, W.-H.; Chen, H.; Lin, M.-C.; Hwang, B.-J.; Dai, H. High-Capacity Rechargeable Li/Cl2 Batteries with Graphite Positive Electrodes. ***Journal of the American Chemical Society*** 2022, 144, 22505-22513.
19. Liu, H.; Chen, C.-Y.; Yang, H.; Wang, Y.; Zou, L.; Wei, Y.-S.; Jiang, J.; Guo, J.; Shi, W.; Xu, Q.; Cheng, P. A Zinc–Dual-Halogen Battery with a Molten Hydrate Electrolyte. ***Adv. Mater.*** 2020, 32, 2004553.
20. Hou, S.; Chen, L.; Fan, X.; Fan, X.; Ji, X.; Wang, B.; Cui, C.; Chen, J.; Yang, C.; Wang, W.; Li, C.; Wang, C. High-energy and low-cost membrane-free chlorine flow battery. ***Nat. Commun.*** 2022, 13, 1281.
